# Toward epigenetic and gene regulation models of specific language impairment: looking for links among growth, genes, and impairments

**DOI:** 10.1186/1866-1955-4-27

**Published:** 2012-11-24

**Authors:** Mabel L Rice

**Affiliations:** 1 University of Kansas, Lawrence, KA, USA

## Abstract

Children with specific language impairment (SLI) are thought to have an inherited form of language impairment that spares other developmental domains. SLI shows strong heritability and recent linkage and association studies have replicated results for candidate genes. Regulatory regions of the genes may be involved. Behavioral growth models of language development of children with SLI reveal that the onset of language is delayed, and the growth trajectories of children with SLI parallel those of younger children without SLI. The rate of language acquisition decelerates in the pre-adolescent period, resulting in immature language levels for the children with SLI that persist into adolescence and beyond. Recent genetic and epigenetic discoveries and models relevant to language impairment are reviewed. T cell regulation of onset, acceleration, and deceleration signaling are described as potential conceptual parallels to the growth timing elements of language acquisition and impairment. A growth signaling disruption (GSD) hypothesis is proposed for SLI, which posits that faulty timing mechanisms at the cellular level, intrinsic to neurocortical functioning essential for language onset and growth regulation, are at the core of the growth outcomes of SLI. The GSD highlights the need to document and account for growth patterns over childhood and suggests needed directions for future investigation.

##  

The remarkable ability of young humans to acquire speech and language, without explicit tutoring, during the preschool years is an unsolved mystery. Debates have swirled around the relative contributions of nature and nurture. Although uniquely linguistic innate abilities have long been assumed by some scholars and challenged by others, until recently explicit genetic investigation was not attempted. A first wave of candidate gene discoveries has been reported recently for language, speech and reading impairments in children and their families. A recent special issue of this journal, published in December 2011, focused on epigenetics for studies of the etiology of language, speech, and reading impairments. Epigenetics, referring to mechanisms ‘above’ the genome, involves modifications of DNA or associated proteins, other than DNA sequence variation, that carry information content during cell division. The epigenome consists of chemical compounds that modify, or mark, the genome in a way that tells it what to do, where to do it, and when to do it. Epigenetic marks, not part of the DNA itself, can be passed on from cell to cell as cells divide, and from one generation to the next [1]. This level of genetics inquiry would be a new direction that could build on and clarify the recent candidate gene discoveries reported for language, speech, and reading impairments, or shift attention to new mechanisms of gene regulation. The collection of papers in the special issue covered the following: the role of epigenetic modifications underlying the developmental consequences of early life events [2], recent candidate gene investigations, possible phenotypic interrelationships, and challenges for interpretation [3-5], a call for phenotypes at the level of neuronal pathways and local brain circuits involved in speech and language processing for the next generation of genetics/epigenetics investigation [6], and how the effects of cochlear implants on deaf children’s language acquisition depend on the age of implant, suggesting possible epigenetic influences [7].

This paper takes up the theme of epigenetics and other possible regulatory genetic mechanisms in the context of the phenotypes associated with specific language impairment (SLI), a condition with genetic influences. Language change over time in children with SLI demonstrates both weaknesses and strengths, tied to age-related phases of language acquisition. The key concept here is the timing of language growth, with suggestions about how regulatory mechanisms, epigenetic and genetic, may provide clarifications of timed genetic effects that play out as delays and long-term deficits in language, in cascades of phenotypic outcomes.

The present review unfolds in five main sections. The first three sections focus on the behavioral phenotypes of SLI. The first section provides a brief description of the speech and language phenotypes associated with children with SLI, with the exclusionary criteria intrinsic to the diagnosis. The second section provides an overview of the ways in which language grows over time in children with and without SLI, and the issues of interpretation that arise from the findings. The third section highlights new questions generated from the growth findings, questions that call to mind possible biological underpinnings of a growth drivetrain for language acquisition. The fourth and fifth sections provide a selective summary of candidate gene discoveries for language impairment, followed by a discussion of epigenetic models relevant to language acquisition and impairment and consideration of possible mechanisms involved in the three elements of language growth in SLI: delayed onset, acceleration, and premature deceleration. A growth signaling dysfunction model of SLI is proposed, with implications for future investigation.

## Review

### Children with specific language impairment

Language impairments in children can appear alongside other developmental diagnoses, such as intellectual impairments, hearing loss, and syndromes such as Williams, fragile X and autism spectrum disorders [8]. More frequently, language impairment is present with no other obvious causes, a condition known as SLI. The best population prevalence estimate for SLI is 7.4% of children at school entry (5 years), defined as language performance more than 1 standard deviation below the mean for the age, roughly in the bottom 15th percentile of the age group, excluding children with low levels of non-verbal intelligence, hearing loss, neurological impairments, clinical level social disorders, autism or other developmental syndromes [9]. In short, children with SLI have the assumed prerequisites for language to emerge on time and unfold as expected, yet the expectations are not met.

The existence of children with SLI can be counter to general expectations of the relationship of language to general cognitive skills or the relationship of language to speech acquisition. With regard to cognition, it is important to note that language acquisition and non-verbal intelligence levels are not fully associated in a causal relationship. Simply put, language can be selectively impaired or selectively spared relative to non-verbal IQ abilities. Because children who have good language skills can often mask low levels of non-verbal intelligence, detection of the dissociation requires epidemiological survey studies of children in the general population. Such evidence was reported in a large scale epidemiological study contracted by the National Institute of Deafness and Communicative Disorders (NIDCD) [9]. At school entry, 12% of children who scored low on non-verbal IQ assessment (in the range of 70 to 87 IQ) demonstrated overall language performance within normal limits [10]. Rice and colleagues [11] found that this group of children with low non-verbal IQ levels and expected general language skills also did not differ from control children in specific grammatical skills; this group used well-formed sentences similar to children of the same age with higher non-verbal IQs. Thus, language can be selectively spared relative to non-verbal IQ abilities. However, children who have both language impairments and non-verbal cognitive impairments scored lower, and grew more slowly in language skills between 6 and 10 years, than children with SLI [11]. In effect, language impairments are not necessarily part of a global cognitive developmental impairment in healthy children but if both language and cognition are at low levels, the combination yields lower language performance than language impairment alone, and slower growth over time.

With regard to speech skills, it is important to differentiate between speech disorders (involving the production of sounds and intelligibility of speech) and language disorders (involving grammar, vocabulary and narrative abilities). Population-ascertained samples of children at school entry yielded an estimated 2% overlap of speech and language impairments [10]. This was rather surprising, given that clinical caseloads show more overlap of speech and language impairments, probably because speech impairments have noticeable impact on intelligibility whereas children with clear speech and language impairments are less likely to be identified. In a population-based sample, only 29% of children with SLI had been enrolled in clinical services, presumably because the children are not identified or referred for services [9]. The point here is that causal models of SLI must be mindful of the probable dissociation with general non-verbal cognitive abilities and the dissociation with speech impairments in the general population of school aged children. Speech impairments are not diagnostic for language impairments.

An early, but inconclusive, indicator of possible SLI can be apparent at 24 months of age. It is well known that some children show late language emergence (LLE) at 24 months with no apparent cause, as documented in a sample of 1,766 epidemiologically ascertained children [12]. A follow up study of these children (N = 128 with LLE, compared to 109 with documented normal language emergence) found that the majority of children with LLE ‘outgrow’ their apparent language impairment by 7 years of age [13]. Nevertheless, the risk of SLI at 7 years of age is higher for children who have a history of LLE, especially in the domain of grammar. Grammar appears to be an especially sensitive measure of the neurocognitive mechanisms needed for language acquisition. A further important finding is that LLE is three times more likely in males than females at 24 months, although the gender differences disappear between 5 and 7 years [13], suggesting a need to keep our eye on potential gender-related effects especially during the early period of development. Parental education levels are likely to be lower for children with SLI than control children. After adjusting for parental educational levels, two other predictors for LLE at 24 months are of interest. One is a history of late language emergence in family members and the other is suboptimal fetal growth [12]. A shared family history of LLE may reflect either genetic or environmental influences. The suboptimal fetal growth is consistent with possible epigenetic influences on birth weight [2]. An earlier case control study of 5-year-old children with SLI and controls [14] reported an association of parental learning histories but no statistically significant perinatal events, including birth weight, or maternal exposures to disease. The difference in outcomes may be due to the difference across the studies in the method of calculating fetal growth at birth. Suboptimal birth weight is calculated with adjustments for maternal factors that influence the child’s birth weight and may be a more sensitive index of prenatal influences on growth [12].

Children with SLI are at high risk for reading impairments in middle childhood. About 50% of young children with SLI have subsequent reading impairments [15]. Also, children with SLI are likely to have siblings or parents with a history of LLE, SLI, and/or reading impairments. Family aggregation studies report about 25% of family members of probands are affected [16]; direct assessment of family members yields estimates of 32% to 48% of the family members as affected based on general language tests [17], with higher rates of affectedness in younger siblings than older siblings or parents. Twin studies yield heritability estimates of 0.25 for SLI [18]. It is also reported that twin heritability estimates can be higher if the diagnosis of SLI is drawn from a clinical population in which speech impairments and low non-verbal intelligence coexist with language impairments, although significant heritability was found only for speech impairments, not language impairments, even if children with low non-verbal IQs were excluded [19].

In summary, although language impairments are at the core of the ways in which children with SLI differ from other children, it is also the case that language impairments are linked to other risks as well, including reading impairments, family members with language impairments and lower academic achievements. Further, although it is beyond the scope of this paper, social development is also affected, in the way of restricted friendship networks and increased risk for identification as mildly introverted [20-22]. Particularly in the early years of school, their teachers often regard the children with SLI as generally ‘immature’. Thus, SLI can appear in complicated clusters with reading impairment, social adjustment issues, and familial risks for language impairment, reading impairments, and low academic achievement. At the same time, SLI can appear in children and families with good reading and social skills and parental professional degrees. The causal pathways are complex, with limited language skills influencing early social skills (as shown by comparisons in preschool classrooms of children with SLI and children learning English as a second language [23,24]) and later school achievement, along with the possibility that these different phenotypes are influenced by shared genetic risk [3,5].

### Change over time: linguistic dimensions, similarities and differences between children with SLI and controls

As argued by Poeppel [6], the search for causal factors, including the role of genetics, depends on the underlying models of language and related neurocognitive systems under genetic influence. Interpretations of the nature of SLI in children vary according to the underlying theories of language. Although the exact assumptions are usually not made explicit, it is fair to say that the prevailing view is something along these lines: language acquisition is driven by general learning mechanisms that act on input processing mechanisms that may or may not be specialized for speech and are stored in memory by processes that may or may not be specialized for verbal information and retrieval. Under this general perspective, the language limitations of children with SLI are ascribed to breakdowns in input processing [25,26], limited verbal memory (a relatively large literature exists in investigation of non-word repetition as an endophenotype of SLI and/or a causal factor in language impairments [27,28]), a limited amount of general language ability such that the children with SLI have less capacity of the same sort as other children, or a breakdown in domain-general implicit learning [29] or statistical learning abilities [30]. This general learning deficit model is compatible with findings showing that if non-verbal intelligence levels are low in children who otherwise meet the diagnostic criteria for SLI, language levels are likely to be lower than in children with SLI whose non-verbal intelligence is within or above normal range. However, the general learning deficit model does not account for the documented cases of children with selective non-verbal intellectual impairment and spared language ability.

An alternative draws on theories of linguistic representations in the adult grammar for hypotheses of underspecification of particular levels of linguistic representations in children with SLI [31-34]. This perspective assumes innate abstract language specific mechanisms that guide language acquisition across the world’s many different languages in interaction with more general learning mechanisms that allow children to learn the forms of their particular language [35]. A large body of replicated empirical evidence about SLI has accumulated, documenting predicted areas of linguistic weaknesses in the grammar of children with SLI, as originally hypothesized by linguistically motivated hypotheses.

Overall, to date, across the wide range of interpretations of the causes of SLI, interpretive consensus is out of reach. A shared challenge for the available models is that none are fully adequate to the challenges of accounting for the recent empirical documentation of the ways in which grammar and vocabulary increase over time in children with and without SLI. Genetic inquiry could help resolve some of the interpretive questions. There is increasing technical documentation of the powerful ways in which genes and gene expression influence development, at the cellular level and also at the cortical level, and how genes and gene expression influence the emergence (and decline) of general cognitive abilities in animal models as well as for human development [2-5,36,37]. Although the biological mechanisms are yielding to experimental methods, there is a large gap in the empirical human evidence base, particularly at the level of detailed longitudinal description of children’s language acquisition over the years encompassing the transition from child language to adult language. In short, documentation is sparse for the important issue of how language grows over time in childhood, especially for how growth plays out for children with SLI.

One longitudinal study in progress (funded by the National Institute for Deafness and Other Communication Disorders, #001803, to the author as principal investigator) provides needed documentation of growth in the age range of 2 to 20 years The study is a family-based candidate gene program of genetic inquiry based on a proband ascertained as SLI (usually ascertained in the age range of 4 to 8 years) with recruitment of parents and siblings, compared to a control group of children and their families. The probands meet the criteria of no hearing loss, non-verbal intelligence levels at a standard score of 85 or above (that is, in normal range or above), no known developmental or neurological disorders, and monolingual speakers of English whose families have stable residency in the local region. The probands also meet a core inclusionary criterion of language performance at a standard score below 85 (roughly, in the bottom 15th percentile of their age group). A control group of children meets the exclusionary criteria at study entry, as well as a language standard score of 86 or above. Children whose language standard scores are above 120 are not included as control children. To date, more than 200 probands with SLI are participating and 90 control children, and families. The current total participant base with longitudinal direct behavioral assessments is more than 900. In this sample the occurrence of SLI in siblings is roughly 25%, consistent with other studies [38]. The probands have been followed in an age range that encompasses 2 to 26 years; the longest series of longitudinal data points for the proband children encompasses 17 years, with most children studied for more than 5 years. Although most of the probands were enrolled in speech/language services at the time of entry into the study, ongoing monitoring of the services they were receiving invariably shows that the children were dropped from these services by mid childhood although, on average, they were likely to receive help with reading in middle childhood. The protocol of assessments included detailed language assessments of overall language skills, grammar and semantics appropriate for age levels, administered every 6 months for children under 9 years and annually for children 9 years and older. The study is unique for the relatively large sample size of probands with SLI; the rigorous criteria for identification of SLI and control for other factors such as low levels of non-verbal cognitive performance or bilingual exposure; the inclusion of direct assessments on family members (including longitudinal assessments of siblings with and without SLI), and controls; distributed ascertainment over a geographic area not limited nor defined by a particular clinical or intervention setting; detailed direct-assessment behavioral protocols on all participants every 6 months or annually; and genotyping and genetic studies running in tandem with the behavioral data collection and analyses.

The database yields growth curves covering the age range of 2 to 20 years, across different dimensions of language acquisition, comparing children with SLI and control children, on actual direct behavioral assessments of the same child assessed over time. The data are reported here as growth trajectories to make it possible to compare across the target dimensions of language across ages. Although earlier technical analyses of the data have been reported, and cited below, the curves reported in the figures here are new for the purpose of comparisons and updates with additional data as it has become available.

There are two elements of the growth curves to note: one is the level at the first time of measurement, and the second is the pattern of change over time (that is, whether change follows a consistent, linear rate of growth or if change is curvilinear, with times of acceleration or deceleration). The language measures are age appropriate, span multiple dimensions of language, and vary over the full age range. For some measures, given the trajectory of change over time, there are clear baseline and ceiling effects defined by the ages at which children can first respond and, at the upper levels, when the control group reaches ceiling levels. The age of appearance of ceiling effects is an empirical outcome of the study. The aim is for a series of tasks that capture age-defined growth patterns. If the growth curves vary per task it is even more noteworthy if the SLI group shows the same patterns of growth, suggesting similar underlying timing of growth, or similar underlying constraints in the language acquisition mechanisms. A long-term empirical aim is the development of valid grammar growth measures encompassing the full age range.

**Figure 1:**
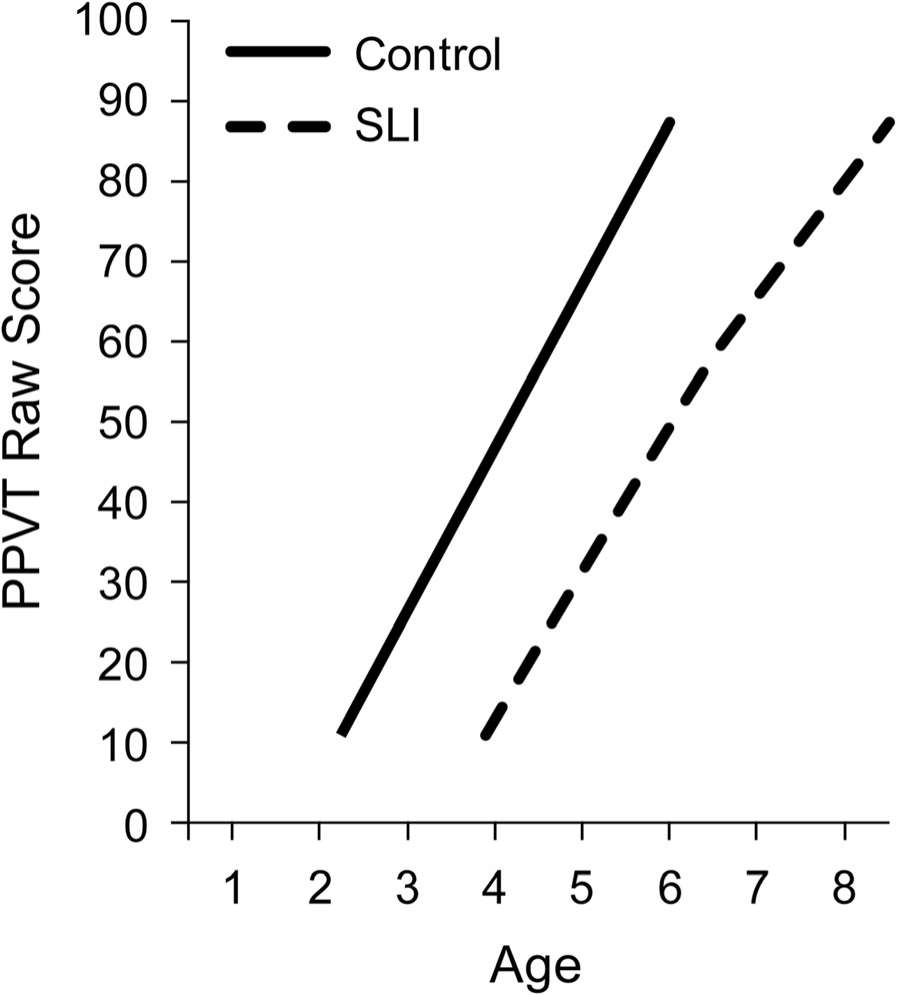
Growth of receptive vocabulary for controls and children with specific language impairment (SLI).

At the youngest ages language emergence was evaluated in a picture identification task to assess early vocabulary, the Peabody Picture Vocabulary Test (PPVT) [39]. Figure 1 displays the PPVT raw scores for control children and children with SLI in the age range of 2 to 8 years (see [40] for longitudinal analyses and [41] for cross-sectional analyses that yield data that replicates the first study although the longitudinal analyses are not reported in the second study). At the outset, the figure documents about a 2-year advantage for the earliest ages in which the control children comprehend words compared to the SLI children. The growth patterns show that once vocabulary comprehension is underway the growth trajectories for the two groups are highly similar. Early growth in vocabulary comprehension is mostly linear, with a relatively steep rate of growth in this age period. Although the SLI group is adding new words to their vocabulary at a brisk rate, as a group they do not close the gap between them and the controls during this age range because the same rate and pattern of change cannot overcome the delayed onset.

**Figure 2:**
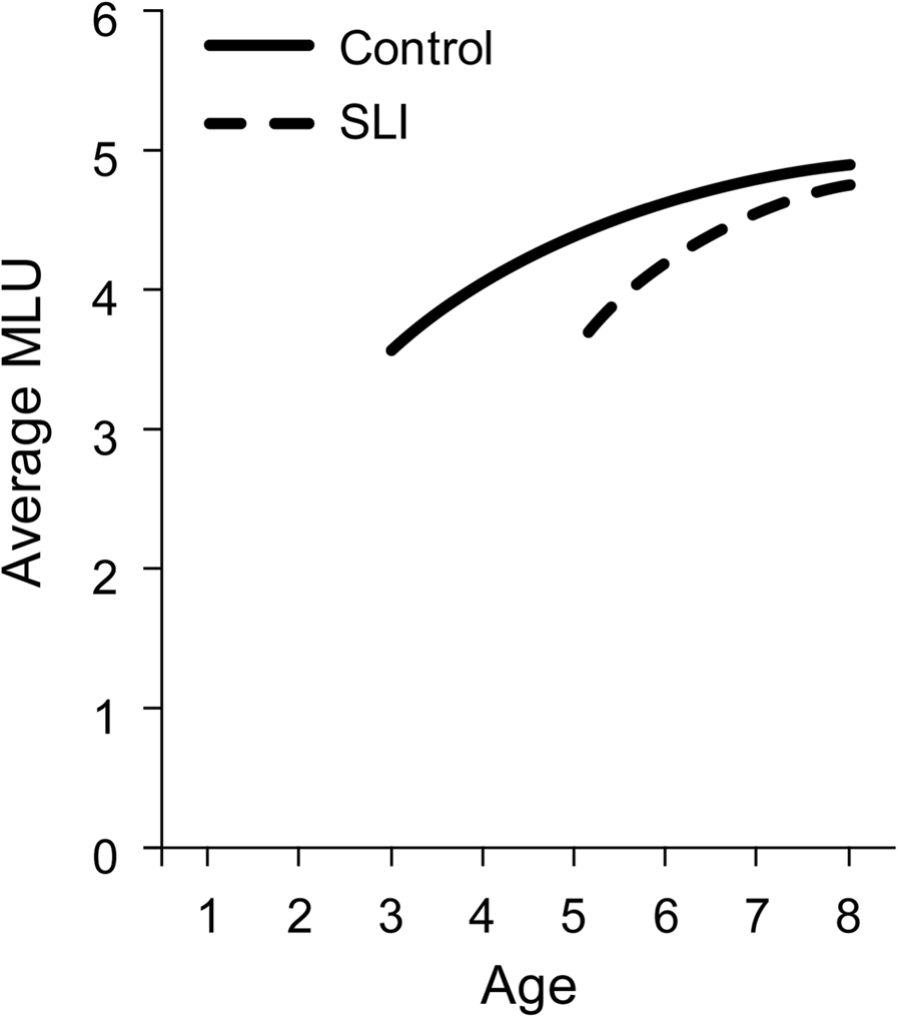
Growth of mean length of utterance for controls and children with specific language impairment (SLI).

Another index of young children’s language growth is their mean length of utterance in their spontaneous utterances. The number of words or morphemes in children’s utterances during play sessions is a valuable indicator of their ability to form sentences, a developmental advance beyond their ability to understand single words [42,43]. Figure 2 shows growth in the period from 3 to 8 years, comparing the SLI group and a control group of children (compare [40] and [41]). For this index, the 2-year difference between groups is also apparent early on, as children begin to combine words. The average utterance length is about 3.5 morphemes for the controls at an average of 3 years whereas the children with SLI on average do not hit that level until 5 years. The growth trajectory is not linear for this index but instead grows quickly to a natural leveling off around five morphemes, a level achieved several years earlier for the controls than for the children in the SLI group. Growth models do not differentiate between the two groups, indicating that both groups follow the same trajectory, with the similarity across groups suggesting that it is likely that both groups are tapping into similar mechanisms of change. Because of the natural leveling for this index, with a resultant deceleration at the upper levels, the SLI group does eventually close the gap for utterance length although it requires several years to do so. For phenotyping purposes, the obvious ceiling effect for this measure limits its use to the early childhood years.

**Figure 3:**
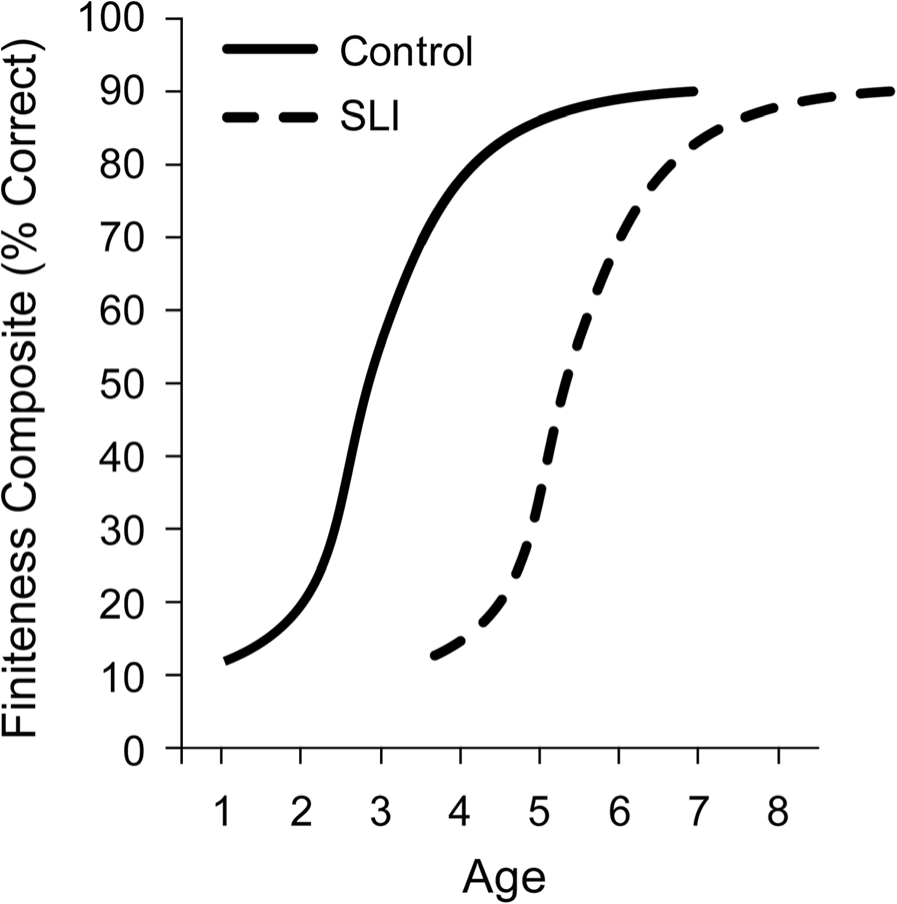
Growth of the percentage of required finiteness markers in the utterances of controls and children with specific language impairment (SLI).

In the next figure the language measure shifts to formal properties of grammar as indicators of ongoing language impairments in children with SLI. Figure 3 reports the percentage of required finiteness markers in children’s utterances. Finiteness marking is evident in English in the use of a small cluster of verbs and verb conjugations. In the following sentences, the finiteness markers are enclosed in parentheses: The boy (is) happy. The girl (is) running. The dog run(s) home. The cat walk(ed) home. You run (ran) away. This property of the language is manifest differently across languages, differences that affect the age of acquisition. For young children acquiring English, early on they are likely to omit finiteness markers, an obligatory property of the adult grammar. This phenotype can be aligned with formal models of the adult grammar and the underlying unifying linguistic constructs involved, for estimates of progress toward the adult grammar and comparisons across ages and groups [44,45]. For children with SLI, this property of the grammar is particularly difficult, lagging behind other indicators of language acquisition. Finiteness markers are evident first in simple declarative clauses used by children and later in children’s questions.

Figure 3 documents growth in finiteness marking in the period of roughly 3 to 8 years in groups of children with and without SLI [11,46-48]. Note that children with SLI are more than 2 years delayed (relative to controls) for this grammatical property, and this greater delay persists for years. Sometimes this weakness in grammar is called a ‘deficit’ because children with SLI perform below younger children at equivalent mean length of utterance (MLU) or vocabulary level, suggesting a selective limitation beyond the general profile of delayed language. Even so, as with the previous figures, once the growth trajectory begins it unfolds in the same way as the control group, reaching a natural asymptote of adult-like competencies in the simple declarative sentences used in the experimental tasks, a level the control group reaches, on average, before 4 years of age but the affected group, on average, does not attain until beyond 6 years. Formal growth models repeatedly confirm that the growth trajectory of the two groups does not differ, but the age of onset does, even though the pattern of growth for this part of the grammar has strong non-linear components with a pronounced acceleration early on and a pronounced deceleration near the adult level of 90% accuracy in required contexts. Again, it is as if the children with SLI are prepared to employ the same growth mechanisms as the control children once the system starts.

**Figure 4:**
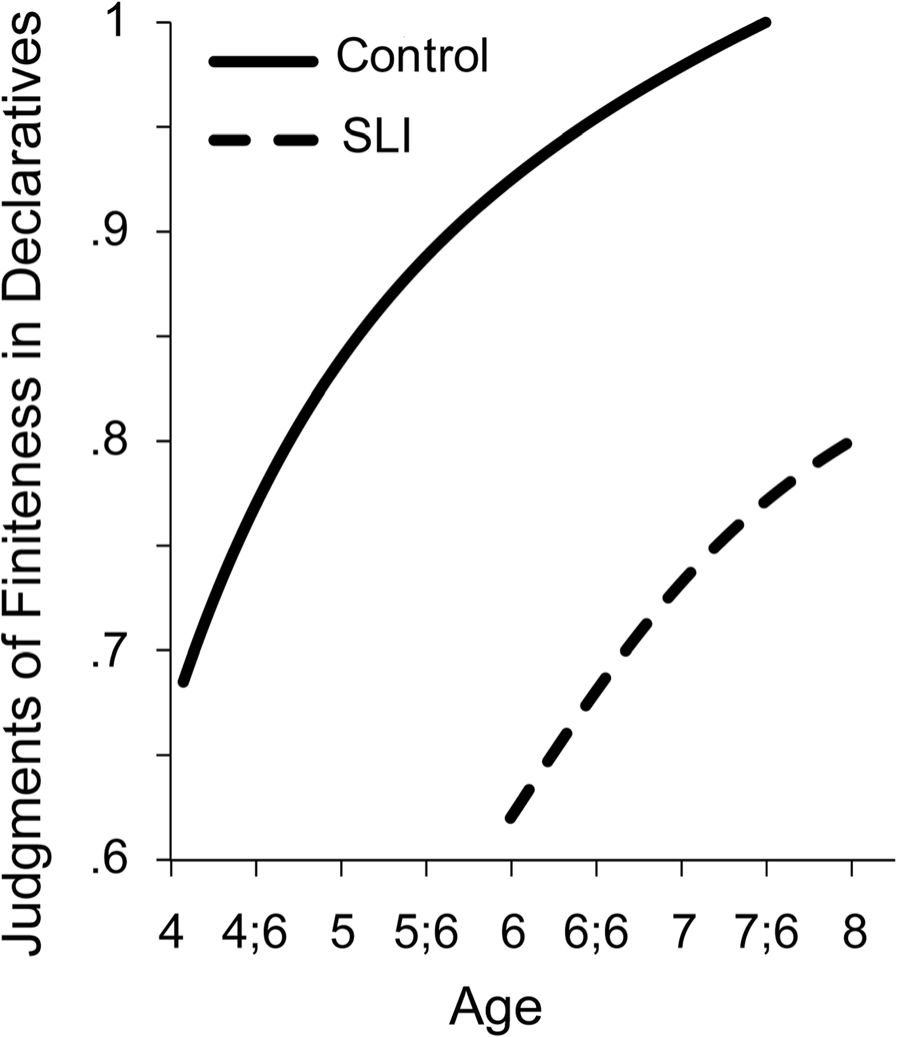
Growth of the accuracy levels in judgments of finiteness marking in simple declarative clauses by controls and children with specific language impairment (SLI).

For continued observation of the finiteness requirement as children grow older, an experimental task was developed that requires the children to make judgments of grammatical well-formedness in a series of sentences similar to the examples in the previous paragraph, with finiteness markers sometimes used and sometimes omitted, a pattern thought to mirror the rules of the underlying grammar for children in the SLI group. Figure 4 reports the growth trajectories of the two groups for an experimental judgment task, for the age range of 4 to 8 years (compare [48] and [49]). Once again, the two groups differ at the age at which they reach performance of 0.7 on the judgment index, separated by more than 2 years, but once started the growth curves do not differ, as confirmed by formal growth analyses. It is noteworthy that for the judgment data we see that the SLI group does not close the gap at 8 years but seems to decelerate around 8 years, suggesting that ultimately they do not reach the accuracy levels expected for the adult grammar.

**Figure 5:**
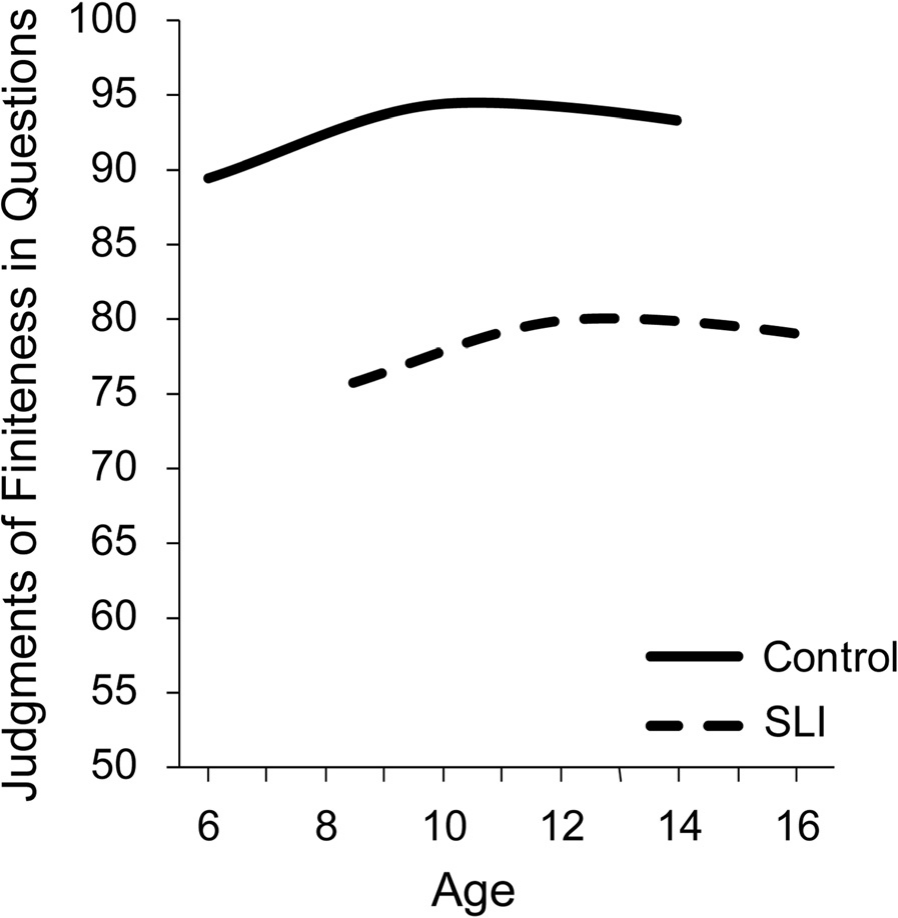
Growth of the accuracy levels in judgments of finiteness marking in simple questions in controls and children with specific language impairment (SLI).

Task difficulty is increased further by shifting from simple declarative sentences to questions, which also require use of copula or auxiliary forms of BE or auxiliary DO in order to have a well formed question, as shown in these examples: Where (does) he go? (Is) she happy? Where (is) he running? (Does) the cat have a home? Figure 5 expands the age range to 16 years and shows that there is little growth for children with SLI from 8 to 16 years on this task, where they are ‘stuck’ around the 0.75 accuracy level, significantly below the 0.90 or above of adult-like performance of the control group [50]. This follows the indication from Figure 4 that if the children with SLI do not ‘catch up’ with their peers by middle childhood they are greatly at risk for closing the gap. Instead, their grammar remains at an immature level into adolescence and the adult period.

**Figure 6:**
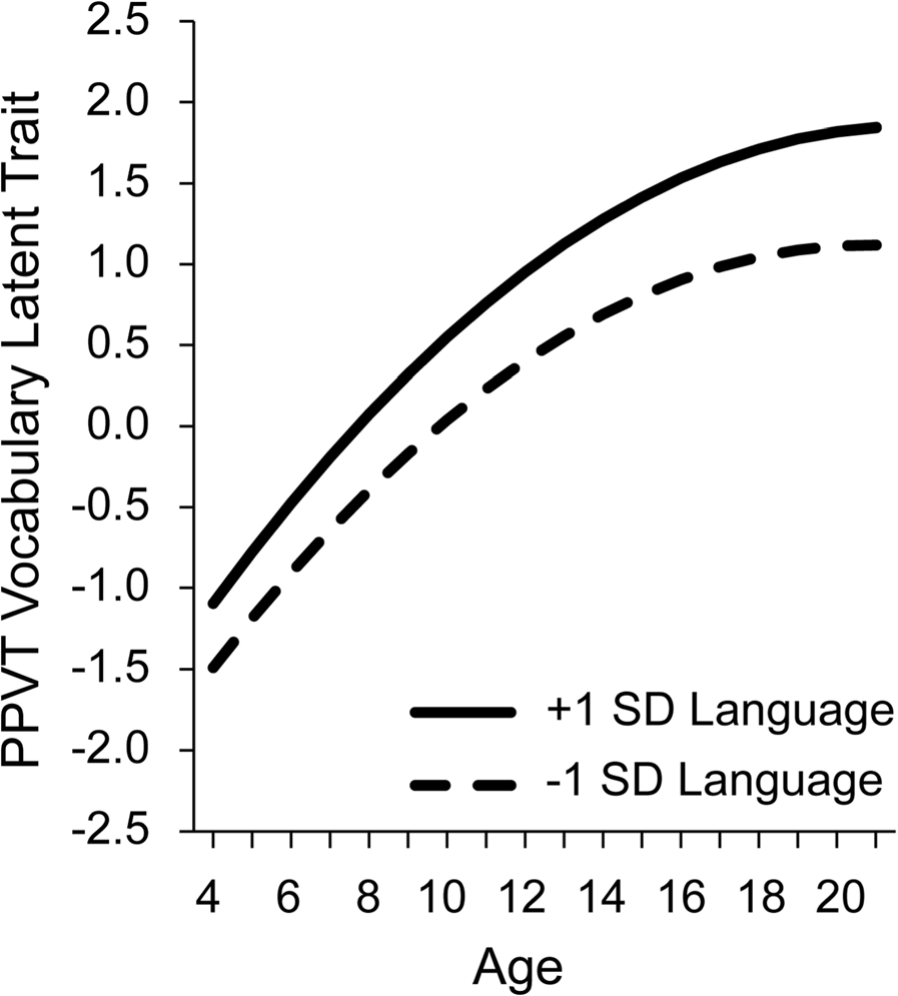
Growth of latent trait receptive vocabulary for controls and children with specific language impairment (SLI).

Leveling at upper childhood is also apparent in vocabulary acquisition. Figure 6 reports preliminary analyses of PPVT receptive vocabulary scores for the age range of 4 to 20 years from the data archive. In these analyses an underlying vocabulary trait index was calculated on the basis of item level responses, allowing for a continuous estimate over time and different versions of the test, similar in interpretation to (although not exactly the same as) the raw scores presented in Figure 1[51]. In Figure 6 the full sample is divided based on scores at the first time of testing into a group of children more than 1 standard deviation above the mean on the trait score, roughly at the 85th percentile or above, compared to a group of children more than 1 standard deviation below the mean, roughly at the 15th percentile or below. The modeled trait scores are centered on age 10 years, with a 0 score at 10 years for the full group. Children younger than 10 are expected to have negative trait estimates (smaller vocabulary) and older children have positive trait (larger vocabulary) estimates. As in the earlier figures, we see that children who perform at low levels follow the same growth trajectories as children who perform at high levels, and formal growth model computations confirm the trajectories of the two groups do not differ. Also note that, as suggested by Figure 5, if the children at low levels do not close the gap and ‘catch up’ by mid childhood, by somehow accelerating at an even faster rate than the children at the higher levels, the low group follows a growth path that keeps them at lower, and leveling off, rates of receptive vocabulary acquisition.

The series of growth trajectories in Figures 1, 2, 3, 4, 5 and 6 provide fine-grained documentation of how it is that children with SLI are likely to become adolescents with SLI [52,53], and to later become parents of probands with SLI, parents with a risk of low language performance [17]. In effect, on average it is unlikely that children with SLI ‘outgrow’ their early language impairments. Essentially, it appears that SLI is a condition with unexplained individual differences in language acquisition, differences that persist over time such that a child with SLI is likely to remain in the lower levels of language performance, relative to age expectations. It is not that the child’s language does not change with time; in fact, the language of children with SLI seems to change in ways very similar to unaffected younger children, across the evolving grammatical and vocabulary levels that document the shift from child to adult grammars. The defining differences seem to be at the outset, with likely delays in onset of the rolling changes in the language system over time, and at expected deceleration in language growth first appearing in mid childhood as some parts of the grammar/grammar processing systems reach the adult levels early on and in later childhood as other elements of the grammar/grammar processing systems reach the adult levels and in early adulthood as elements of the vocabulary/vocabulary processing systems reach adult levels. The children with SLI seem to master the basics of language acquisition, with clear speech productions, by middle childhood, such that their ongoing linguistic weaknesses are likely to be undetected in casual conversational interactions. At the same time, children with SLI, and their parents with similar histories who participate in our studies, tell us often that they know they are not good at grammar tasks and related areas of schoolwork, no matter how hard they tried to master the tasks that come easily to most children.

### How to account for new facts about language growth in children with SLI?

The growth data generate new questions. Perhaps most impressive is the clear evidence that, across different kinds of language tasks, the acceleration of language performance is as robust, once it starts, in children with SLI as in children without SLI. The acquisition mechanisms are engaged, and follow the same growth trajectories, even as the particular linguistic elements follow different trajectories, some of which are mostly linear and some are curvilinear with strong shifts in acceleration and deceleration. Further, up until age 8 years or so the children with SLI arrive at the uppermost levels of performance although at an older age than that of the children without SLI. The unmistakable conclusion is that acquisition mechanisms show surprising strengths in children with SLI, particularly in early childhood.

Overall, the generalization is that language performance is likely to persist at a level below age expectations into the adult age range. The outcome appears attributable to a delayed onset at the outset. Language acquisition can be compared to a train with an expected time of departure, a particular path to follow, an expected rate of acceleration and speed along the path. The language of children with SLI seems to have a delayed departure but a similar path and speed once underway [54]. Their language train, however, seems to decelerate before reaching the final destination of the adult grammar, not quite reaching the end goal.

These facts strain the scope of existing causal models. Models that posit a weakness in general learning mechanisms cannot account for how children with SLI achieve such robust growth in spite of limited learning mechanisms. Likewise, models that posit a breakdown or weaknesses in input processing mechanisms specific to speech or verbal memory limitations leave us wondering how the children are able to overcome those limitations to acquire grammatical distinctions or new vocabulary in the same amount of time as younger unaffected children once the systems begin to show change, in the same growth patterns over time. Models based on linguistic representations are supported in the expectation that change is not the same across different dimensions of language, and the prediction that finiteness (in English) brings particular challenges to children, and especially so for children with SLI. Yet linguistically motivated models also hypothesize learning processes, but offer no specific account of the growth trajectories observed. A recent critique of computational models, as applied to the grammar of typically developing children, highlights the limitations of current models [45]. There are no computational models known to this author that model linguistic growth of children with SLI.

Let us consider the importance of the fact that the strong parallels to the growth trajectories of children without SLI also make it unlikely that the children with SLI ‘catch up’ to unaffected children. In order to accomplish that outcome, the affected children would have to change faster than the unaffected children. This generalization holds for a broad age range, encompassing roughly 5 to 10 or 12 years of age. The exception is in the early period of language acquisition, roughly 2 to 5 or 7 years of age, when ‘catch up’ is attested [13], after which the children with accelerated growth then seem to adopt the same growth curves as other children. The mechanisms for accomplishing an increased acceleration in a short time interval early in childhood are not known, nor are the mechanisms that apparently reset the acceleration to expected levels. Further, there is no adequate model accounting for the variety of expected growth trajectories differing by linguistic dimension but shared by the groups.

Language growth, per se, does not seem to be the central issue in the challenges facing children with SLI. Instead, the first challenge is at the outset, where the onset of growth is delayed, leading to a cascade of delays across different elements of the linguistic system as the youngster ages into the child grammar and vocabulary that then move on toward the end-state adult grammar and vocabulary levels. A late start at the beginning, LLE, is a well known, although incompletely understood phenomenon. However, the second challenge at mid childhood is seldom documented. This challenge is that the available time for sustaining the growth trajectories can ‘run out’, with a deceleration phase that levels off before the end-state adult grammar is achieved. The data summarized here are, as far as this author is aware, the first documentation of the actual deceleration curve for language acquisition in the particular domains measured. The deceleration is most evident in the finiteness element of the grammar, among the variables reported here, but also is evident in the vocabulary domain as well. It appears as if changes associated with pre-adolescence or early adolescence age levels set the stage for decreased language acquisition, leveling off the momentum toward the adult grammar and leaving the system only partially attained. This corresponds to the possibility of a diminished ability to learn second language by early adolescence, a controversial but well documented phenomenon [55]. In the context of family-based genetic studies the deceleration and leveling of language performance below expected adult levels means that affected parents can be detected via direct assessment.

### Exploring genetic etiology of SLI: candidate gene discoveries

Genetic models could help fill in the gaps in the existing accounts of the etiology of SLI. In the past 5 years an initial wave of candidate gene studies of SLI has been reported with encouraging outcomes. The findings are summarized in detail in the December 2011 special issue of this journal (see [3-5]). Although this field of inquiry is at early stages, a handful of genes show promise, with more evidence available for dyslexia/reading disability than for SLI/language impairments. Two genes are of particular note, both of which are regulatory genes that influence the functions of other genes. One is the candidate gene, *KIAA0319*, with two studies replicating associations of single nucleotide polymorphisms (SNPs) with language impairment phenotypes in different samples of children and families [17,56]. Although the replication is encouraging, the relatively low effect sizes with relatively small sample sizes call for further replication. At the same time *KIAA0319* has particularly interesting properties. The gene is reported to influence neuronal migration in embryonic rat brain, suggesting possibly similar effects in humans that remain to be established. Further, the putative role of the gene in the etiology of reading disability led to a study of possible regions of epigenetic effects, yielding a region of acetylation that could regulate the effects of other genes, thereby providing further support for a possible regulatory role for this gene [57]. Although this chain of evidence does not establish regulatory gene effects as part of the etiology of SLI, it supports the plausibility of such a chain. A second gene of interest is *CNTNAP2*. This gene is a downstream target of *FOXP2*, a gene implicated in severe and rare forms of language impairment [58]. Variants of *CNTNAP2* are significantly associated with a non-word repetition task, which in turn is associated with SLI in behavioral assessments [59]. More recently, *CNTNAP2* variants yielded significant associations of SNPs with early language development in the general population [60]. The authors caution that the common *CNTNAP2* variants are not sufficient by themselves to account for language and communication disorders in children, but may have more marked consequences when they occur in concert with other genetic or environmental risk factors. *CNTNAP2* is also known to be involved with early neuronal development [61].

### Toward epigenetic and gene regulation models as linkages between growth and SLI

The candidate gene studies add to the evidence from twin studies and family aggregation to support the likelihood of inherited influences on SLI. The outcomes that focus on regulatory genes, such as *KIAA0319* and *FOXP2*, also suggest potentially complex interactions among genes along the causal pathway. Smith [3] concludes that ‘despite replicated evidence for association of single nucleotide polymorphisms within and around the genes, very few coding mutations have been reported to account for their influence on these disorders. This has led to the hypothesis that mutations affecting reading and related disorders are likely to be in regulatory regions’ [62]. These regions and functions regulate the temporal and spatial patterns of gene expression, suggesting possible clues about the temporal dimension of the biological drivetrain for language acquisition.

At the cellular level the mechanisms involved in developmental changes in gene expression are complex and highly interactive. Epigenetic models have appeared recently as accounts of age-related susceptibility to human disease. Feinberg [63] contrasts epigenetic models and genetic variant disease models, with a call for an interactive model. The definition of epigenetics is ‘modifications of DNA or associated proteins, other than DNA sequence variation, that carry information content during cell division’. The two epigenetic mechanisms best understood are DNA methylation (a covalent addition of a methyl (CH_3_) group to the nucleotide cytosine) and chromatin modification of the histone proteins that make up the nucleosomes around which the DNA double helix is coiled. Feinberg and colleagues [64] proposed a common disease genetic and epigenetic (CDGE) hypothesis in which genetic variants could interact with environmental factors related to nutrition, modifying epigenetic marks on the DNA or chromatin, or the epigenome could interact with the genome indirectly, such as variants in gene sequencing that could act on disease susceptibility by affecting the fidelity of the DNA methylation machinery. The central assumption is that epigenetic disease involves disruption of normal phenotypic plasticity. ‘Just as epigenetic change is at the heart of normal development, so also do disruptions in epigenetic modification disturb normal developmental programs’ [63].

Another recent model proposes an epigenetic code in the central nervous system that mediates synaptic plasticity, learning, and memory. Day and Sweatt [36] propose that epigenetic modifications play a role in the central nervous system, ‘initiating functional consequences with a cell or a circuit by modulating gene expression with intracellular signaling cascades’ and ‘activation of critical transcription factors that bind to specific sequences in gene promoter regions’. Their model is at the cross-section of developmental biology and cognitive neuroscience, with an explication of the biological underpinnings of neuronal plasticity necessary for behavioral memory formation, including epigenetic mechanisms that are involved in the regulation of gene expression. They propose that aberrant epigenetic modifications may lead to cognitive disorders that affect learning and memory, in disorders such as Angelman syndrome, fragile X mental retardation, and Rett syndrome, and in neurodevelopmental disorders of aging such as Alzheimer’s disease.

Epigenetic controls are not necessarily the deciding factor for developmental changes in gene expression. There are many other regulatory mechanisms in the cell that govern the synchronized timing of gene expression, to ensure that cellular level developmental processes begin on time but are not active too long, that maintain or suppress plasticity, modulate age effects, suppress harmful genes, and other complex interactions. These include promoters (DNA that initiate transcription of a gene), enhancers (DNA bound with proteins that enhance transcription levels of genes), silencers (DNA sequence to bind transcription repressors) and insulators (prevention of promiscuous gene regulation by nearby enhancers or silencers) [65,66]. Regulatory genes are involved in these processes, as well as interactions between genes or groups of related genes.

Investigation of the ways in which regulatory mechanisms at the cellular level influence higher cognitive processes are just beginning. Early models provide precedents that could be relevant to the etiology of SLI, by shifting attention to possible biological underpinnings of the timed drivetrain underlying language acquisition and impairment. The behavioral growth trajectories identify three crucial stages to be accounted for: (1) at the outset, the neurocognitive infrastructure necessary to activate the onset of language acquisition; (2) the acceleration mechanisms needed to drive and sustain growth over the years of language acquisition; and (3) the deceleration mechanisms needed to account for a general leveling of language growth in adolescence and adulthood. In the interest of simplification here the differences in how growth appears across the dimensions of language will not be addressed, nor will the declines in language abilities associated with aging, some of which are likely to be part of the decline in memory studied by Day and Sweatt [36].

At onset, there are multiple sources of evidence suggesting that the neurological infrastructure for language is under genetic influence. The most direct evidence is from studies of *CNTNAP2*, which codes for a structural protein: contactin-associated-protein-like-2, which is a member of the neurexin family of proteins. The first report of *CNTNAP2* as an autism susceptibility gene was in a study of children with autism [67] in which the parents reported the age at which their child spoke their first word, which served as the phenotype for the association analysis of single nucleotide polymorphisms (SNPs). In this study sex was an important factor for the association signal, with male-male affected sib pairs accounting for effects. The conclusion was that the common *CNTNAP2* risk allele increases the risk for language delay. An independent analysis of children with SLI, but not autism, identified association of *CNTNAP2* variants with lower levels of language ability [59]. Furthermore, *CNTNAP2* variants were associated with early language acquisition, at 24 months of age, in a general population sample [60]. This is consistent with findings that *CNTNAP2* is enriched in brain circuits important for language development [68]. Thus there is growing evidence that *CNTNAP* variants are likely to be active in the development of the neurocognitive infrastructure of language onset. Further, *CNTNAP2* is directly regulated by *FOXP2*, a transcription factor mutated in rare monogenic forms of speech and language disorder [69], suggesting potential breakdowns in timing mechanisms somewhere in the pathway of gene expression necessary for language onset in young children.

A similar interpretation appears in a study of *FOXP2* in persons with developmental verbal dyslexia, in which the authors hypothesize that ‘…proper dosage of *FOXP2* at precise times and locations (at the cellular and tissue levels) is required for normal development’ [70]. The authors also call for examination of the entire >600 kb *FOXP2* locus, with its many transcripts. A further caution is that some isoforms of *FOXP2* may not be transcription factors. Both *FOXP2* and *CNTNAP2* are large and complex genes with numerous isoforms, although the molecular interpretations are limited to specific isoforms of the genes and tissues easiest to work with. The size and complexity of the two genes pose significant challenges for discovery of particular pathways of gene expression implicated in language onset.

Other environmental factors are implicated in the causal chain. Hormonal and nutritional influences are considered as environmental influences in epigenetics. Sex effects are evident at onset, with greater risk for late onset for males [12]; males account for the *CNTNAP2* variants’ associations with autism age at first words [67]; and males with higher levels of umbilical cord blood testosterone were at increased risk for language delay during the first 3 years of life, although testosterone increase may be a protective factor for females [71] Nutritional risks are reported for language impairments in childhood. Maternal use of folic acid supplements in pregnancy, within the interval from 4 weeks before to 8 weeks after conception, was associated with a reduced risk of severe language delay at 3 years of age, defined as only one-word or unintelligible utterances [72]. Nutrition at the time of conception is known to have effects on fetal development that can lead to deleterious outcomes in infancy, childhood, or adulthood [2].

Bloomfield [2] summarizes the consequences of the sequences of genetic and epigenetic mechanisms in play very early in development, cellular level events that depend on well sequenced ‘turn ons’ and ‘turn offs’ involving gene regulations in prenatal development that play out to increase the risk of adverse neurodevelopmental and metabolic outcomes in childhood and later life. Profound epigenetic modifications to the genome can occur in the early embryo, even prior to implantation in the uterine wall [73]. These discoveries help clarify the effects of maternal nutrition during pregnancy, multiple births or late preterm birth on development in childhood and increased health risks in adulthood. At the other end of development, Day and Sweatt [36] propose an epigenetic model of memory decline with age for humans and evaluated in animal models, anchored in evidence that changes in DNA methylation contribute to memory formation and maintenance, and may contribute to memory decline with aging or disease in humans. They also argue that ‘cellular development and cognitive memory processes are not just analogous but are homologous at the molecular level’.

The CDGE hypothesis provides an overarching model of how developmental programs can be disrupted [64]. Epigenetic effects could act in concert with the regulatory mechanisms of *FOXP2* and *CNTNAP2*, or genes with similar functions, for the development of neural pathways essential for the onset of language acquisition, in a sequence of timed chemical signals for cellular functions that account for normal phenotypic plasticity. A disruption in signaling sequences could lead to a timing delay for the onset of language. Delayed onset does not entail disruption in the mechanisms that drive growth after language acquisition begins. The growth curve evidence from children with SLI indicates growth patterns after onset parallel those of younger unaffected children. It appears that once the neural infrastructure for onset is in place, the language system proceeds at an expected pace, until deceleration appears.

Neural sources for the acceleration and deceleration mechanisms evident in language growth trajectories are unknown. Investigations of the immune system provide a model of how acceleration and deceleration could be regulated. Recent advances in cancer treatment are attributable to an insight about the functioning of T cells, a type of white blood cell that destroys infected cells that are recognized as foreign. The crucial discovery was that T cells require two signals to attack a target effectively: one that functions as an ‘ignition switch’ and another as a ‘gas pedal’. As described in Sharma et al. [74], these two signals are in a timed relationship with a third signal that eventually inhibits T cell activity. The sequence begins with stimulation of the antigen receptor (T cell receptor (TCR)) with major histocompatibility complex (MHC) molecules. A second signal is needed for productive activation, mediated by the binding of CD28 on the T cell surface to B7 proteins on the antigen-presenting cell. The combined effect of these two signals is activation of T cells, proliferation, acquisition of effector functions and cell migration. The sequence includes a cumulative braking function. TCR signaling induces the production of the CD28 homologue CTLA4, a T cell-specific molecule with a higher binding affinity for B7, thereby outcompeting CD28 and eventually inhibiting T cell activity. This sequence of signals implies differentiated timing mechanisms, one signaling ‘turn on’, followed by a second signal to ‘accelerate’ a function when needed, and a braking function built into the mechanism that leads to the restriction of T cell activity in order to minimize damage to normal tissues. In the case of cancer treatment, a misunderstanding of the distinction between activation and acceleration mechanisms can lead to disastrous decision making for treatment [74,75].

In the case of growth trajectories of children with and without SLI, the T cell signaling distinctions have clear parallels to the behavioral phenotype evidence and provide a glimpse into how the growth infrastructure could operate albeit on a longer timeline. The simplest model would be a form of delayed onset signaling, but this model is inconsistent with the evidence that most children with late language emergence ‘reset’ their language growth in ways to ‘catch up’ to other children by school age. Late-starting non-SLI children overcome a late start but the children with SLI do not, suggesting a difference in acceleration plasticity. After onset of language, children with SLI seem to be working with a language acceleration function like younger children, with an intrinsic deceleration mechanism that results in premature ceiling effects with language levels below expected adult mastery.

The overall picture of SLI is consistent with a hypothesized growth signaling dysfunction (GSD), involving a dysfunction in the synchronization of onset, growth, and deceleration of language acquisition and underlying neurocognitive circuits, that includes delayed onset, growth parallels to younger children, and risk for immature grammar in adulthood. Normal phenotypic plasticity could be disrupted in cortical neural pathways such that the infrastructure for language onset is delayed and then the growth mechanisms lack the plasticity to reset the acceleration to compensate for the late start or for the premature deceleration.

The GSD model directs attention to the need for the following: a better understanding of the epigenetic and genetic regulation mechanisms of the prenatal and perinatal period related to the emergence of language in infants and toddlers; prospective language acquisition studies that document growth trajectories, possible sex differences, and the role of possible predictive factors, including testosterone and nutritional variables such as folic acid; the development of growth phenotypes to complement the current use of phenotypes collapsed over age levels, in order to investigate onset and acceleration indices as well as the age at which deceleration appears; exploration of methylation and acetylation levels as an index of possible epigenetic effects; identification of regulatory pathways at the cellular level such as the important documentation of possible *FOXP2*-*CNTNAP2* pathways and possible direct effects on neuronal development in brain circuitry used in human language; candidate gene investigations to identify other potential regulatory gene pathways; recognition that the causal chain of SLI probably includes inherited maturational elements that have elements of strength as well as limitations that play out in complex interactions within families; and alignment of intervention goals to language developmental levels such that intervention capitalizes on the natural momentum of a child’s language growth for a particular linguistic dimension at a given time.

## Conclusions

Children with and without SLI and their families demonstrate the rich and robust nature of language acquisition throughout childhood. Start up can be vulnerable to delays in onset but the acquisition patterns across different dimensions of language appear to be surprisingly robust for children with SLI although their actual levels of performance do not, on average, close the gap with their unaffected peers. Although childhood provides strong mechanisms in support of language acquisition, as childhood shifts to adolescence the period of acquisition levels off, leaving the group of children with SLI with lower levels of language acquisition as they move into and beyond adolescence. Current models of language acquisition are not sufficient to account for the growth evidence. Early genetic and epigenetic findings show promise for revealing the biological underpinnings of the growth drive train. A GSD hypothesis is proposed as an account of the language phenotype growth strengths and dysfunctions from toddlerhood to late adolescence. The GSD highlights the need to document and account for growth patterns over childhood and suggests needed directions for future investigation.

## Competing interests

The author declares that she has no competing interests.
